# Sensitive and Specific Detection of Platelet-Derived and Tissue Factor–Positive Extracellular Vesicles in Plasma Using Solid-Phase Proximity Ligation Assay

**DOI:** 10.1055/s-0038-1667204

**Published:** 2018-07-27

**Authors:** Åsa Thulin, Junhong Yan, Mikael Åberg, Christina Christersson, Masood Kamali-Moghaddam, Agneta Siegbahn

**Affiliations:** 1 Department of Medical Sciences, Clinical Chemistry and Science for Life Laboratory, Uppsala University, Uppsala, Sweden; 2 Department of Immunology, Genetics and Pathology, Science for Life Laboratory, Uppsala University, Uppsala, Sweden; 3 Department of Biomedical Engineering, Institute for Complex Molecular Systems, Eindhoven University of Technology, Eindhoven, The Netherlands; 4 Department of Medical Sciences, Cardiology, Uppsala University, Uppsala, Sweden

**Keywords:** microvesicles, myocardial infarction, cardiovascular diseases, vascular homeostasis

## Abstract

Extracellular vesicles (EVs) derived from blood cells are promising biomarkers for various diseases. However, they are difficult to measure accurately in plasma due to their small size. Here, we demonstrate that platelet-derived EVs in plasma can be measured using solid-phase proximity ligation assay with high sensitivity and specificity using very small sample volume of biological materials. The results correlate well with high-sensitivity flow cytometry with the difference that the smallest EVs are detected. Briefly, the EVs are first captured on a solid phase, using lactadherin binding, and detection requires recognition with two antibodies followed by qPCR. The assay, using cholera toxin subunit-B or lactadherin as capture agents, also allowed detection of the more rare population of tissue factor (TF)-positive EVs at a concentration similar to sensitive TF activity assays. Thus, this assay can detect different types of EVs with high specificity and sensitivity, and has the potential to be an attractive alternative to flow cytometric analysis of preclinical and clinical samples. Improved techniques for measuring EVs in plasma will hopefully contribute to the understanding of their role in several diseases.

## Introduction

Extracellular vesicles (EVs) constitute a heterogeneous population ranging from 0.03 to 1 µm in size that are released from cells either by membrane budding (microvesicles) or by exocytosis of intracellular multivesicular bodies (exosomes). 1 2 Exosomes can also be released by secretory autophagy with and without membrane fusion. 3 4 5 The release of EVs is induced by cellular activation, injury, or stress, and their functions vary broadly depending on the source, the activation state of the parental cell, and the generation process. 1 6 This results in EV populations with different content and surface antigens that disseminate the functions of the parental cell and makes it possible to detect and characterize them. 7 8 EVs are involved in many physiological processes such as coagulation, vascular homeostasis, and intercellular transfer of biological messengers such as miRNA and lipids. 8 9 10 11 Elevated levels of EVs are found in many disorders, including cancer and immunological and cardiovascular diseases, which renders them a high interest for clinical assessment. 1 6 9 10 11 12 13 14 15 The phospholipid membranes of the cell surface–derived EVs are also often rich in negatively charged phosphatidylserine (PS) in the outer leaflet and provide a procoagulant surface that contributes to the hypercoagulability observed in many diseases. 16 17

Tissue factor (TF) is the main initiator of the coagulation cascade in vivo. 18 19 This 47-kDa cell surface–bound protein is expressed in tissues surrounding vessels and initiates clotting after an injury by the binding and activation of FVII to FVIIa. TF expression can also be induced in vascular cells, mainly monocytes, which contributes to thrombus propagation in vivo. 16 20 21 22 Beyond initiation of coagulation, the formation of the TF/FVIIa complex initiates intracellular signaling which contributes to the progression of cancer, diabetes, and acute coronary syndromes by regulating inflammation, cell motility, cell survival, and angiogenesis. 23 24 25 TF in plasma is mostly found on EVs, 26 27 and this pool of TF is also most likely to be procoagulant due to the presence of negatively charged phospholipids providing a surface for activation of the coagulation cascade. 28

The most commonly used technique to study EVs in clinical samples is flow cytometry, mainly due to the possibility to rapidly analyze a range of EV populations using multicolor labeling. However, using this technique in particular small EVs with low protein levels on the surface are difficult to measure accurately. They often fall under the detection limit for forward and side scatter 12 29 and have weak fluorescent signals. The true events might also be confused by the presence of antibody aggregates formed in the antibody reagents and centrifugation of antibodies and buffer is required prior to staining to diminish this problem. 30 Moreover, protocols for analysis of EVs in plasma most often include washing procedures that can lead to the loss of small EVs and the occurrence of centrifugation-induced artifacts. 31 32 Whole blood and plasma analysis may on the other hand increase the background noise that may complicate the analysis. Thus, the small size and the low protein levels on EVs make measurement challenging and the lack of sensitive and standardized methods may have slowed the implementation of EVs as clinical biomarkers. Newer instruments and technical development have yielded better resolution for EVs and improvements of flow cytometry and other techniques will be valuable for the understanding of EVs in various diseases. 33 34 35

The solid-phase proximity ligation assay (SP-PLA) that relies on at least triple recognition of the target vesicle/protein and with real-time PCR quantification has been demonstrated to have excellent sensitivity and specificity for protein detection in solutions. 36 37 Previously, it has been demonstrated that cholera toxin subunit-B (CT-B, binding to ganglioside GM1) 38 and Annexin V (binding to PS) capture different populations of EVs in plasma. 39 In contrast to Annexin V, lactadherin is known to bind PS independently of Ca ^2+^ . 40 We therefore established a SP-PLA method to specifically capture and detect PS-positive populations of platelet-derived EVs in small amounts of plasma using lactadherin as capture agent. In addition, we used CT-B to capture EVs with ganglioside GM1. These populations were then analyzed with proximity ligation for CD41/CD61 or TF antigen. We demonstrate here that platelet-derived EVs (CD41 + /CD61+ and PS + ) captured with lactadherin, but not CT-B, are detected and quantified by SP-PLA, and that these measurements correlate well with high-sensitivity flow cytometry. The SP-PLA also allowed detection of EVs smaller than 0.2 µm. TF-positive EVs were detected using SP-PLA with CT-B capture, but not lactadherin, from LPS and TRAP-stimulated whole blood from healthy individuals. However, SP-PLA with lactadherin capture allows detection of TF-positive EVs spiked into plasma with a similar sensitivity as a direct TF procoagulant activity (PCA) assay.

## Methods

### Clinical Samples and Ethical Permission

Plasma samples from 17 patients with ST-elevation myocardial infarction from the Relevance of Biomarkers for Future Risk of Thromboembolic Events in UnSelected Post-myocardial Infarction patients (REBUS) trial collected 3 to 5 days after the index event and at 1 year follow-up 41 were analyzed for platelet-derived EVs. The study was approved by the Uppsala University local ethics committee (Dnr 2009/210). Plasma samples from healthy individuals were drawn to study the generation of EVs by in vitro stimulation of whole blood and the study was approved by the Uppsala University local ethics committee (Dnr 2013/116).

### Blood Sampling and Preparation of Platelet-Free and EV-Free Plasma

Blood was drawn from healthy individuals using vacutainers with 3.2% citrate. Whole blood was then stimulated with 20 µM adenosine diphosphate (ADP), 10 µM thrombin receptor activating peptide (TRAP), or 20 ng/mL lipopolysaccharide (LPS) during 3 hours to generate EVs. Samples were subsequently centrifuged at 2,500 × *g* for 15 minutes and the platelet-poor plasma (PPP) was transferred to new tubes. The PPP was then centrifuged at 2,500 × *g* for 15 minutes again and the platelet-free plasma (PFP) was aliquoted and frozen at −70°C. Repeated freeze–thaw cycles were avoided. The patient samples were centrifuged at 2,000 × *g* for 15 minutes and aliquots were frozen at −70°C. After thawing, samples were centrifuged again at 2,500 × *g* for 15 minutes.

To prepare EV-free plasma, PPP from 10 individuals was pooled and centrifuged at 100,000 × *g* for 18 hours at 4°C. After centrifugation, EV-free plasma was aliquoted and frozen at −70°C.

### Purification of Platelet-Derived EVs

Blood was drawn from healthy individuals using vacutainers with 3.2% citrate and was centrifuged at 140 × *g* for 20 minutes and platelet-rich plasma (PRP) was transferred to new tubes. PRP was stimulated using 20 μM ADP and 10 μM TRAP and incubated at 37°C for 2 hours with agitation every half an hour. After incubation, PRP was centrifuged at 2,500 × *g* for 15 minutes twice to remove any residual platelets. The plasma was thereafter centrifuged for 60 minutes at 20,000 × *g* and the plasma was subsequently removed. The pellet was dissolved using 200 µL HEPES-buffered saline (HBS), and aliquots of 20 μL were frozen at −70°C.

### Antibodies and Reagents

Antibodies used in SP-PLA were mouse antihuman CD41 (555465), mouse antihuman CD61 (555752), and mouse IgG1 (557273) from BD Pharmingen (San Jose, California, United States). Goat anti-TF polyclonal antibody (AF2339) and goat IgG (AB-108-C) were from R&D Systems (Minneapolis, Minnesota, United States). Antibodies for flow cytometry were anti-CD41 (6607115) from Beckman Coulter (Fullerton, California, United States). Lactadherin-FITC (Haematologic Technologies Inc., Essex Junction, Vermont, United States) or Annexin V – Pacific blue (Biolegend, San Diego, California, United States) was used to detect PS-positive EVs. Goat IgG for blocking buffer was purchased from R&D Systems.

### Biotinylation of Lactadherin

Bovine lactadherin was biotinylated using the EZ-Link Micro NHS-PEG _4_ -Biotinylation kit (Thermo Fisher Scientific, Waltham, Massachusetts, United States) according to instructions from the manufacturer. Fifty-fold excess NHS-PEG _4_ was added to lactadherin and the mixture was incubated for 30 minutes at room temperature. Zeba spin desalting columns were used to remove unreacted biotin. Aliquots of 2 µM biotinylated lactadherin were frozen at −70°C until use.

### DNA Oligonucleotides, Primers, and Splint Sequences

DNA oligonucleotides 14 for preparation of PLA probes were modified with a thiol group at the 5′ or 3′ end (5′ SH-CGCATCGCCCTTGGACTACGACTGACGAACCGCTTTGCCTGACTGATCGCTAAATCGTG 3′ OH) and (5′ P-TCGTGTCTAAAGTCCGTTACCTTGATTCCCCTAACCCTCTTGAAAAATTCGGCATCGGTGA-3′ SH), the latter oligonucleotide having a 5′-phosphate group (Eurogentech). Forward (5′-CATCGCCCTTGGACTACGA-3′) and reverse (5′-GGGAATCAAGGTAACGGACTTTAG-3′) primers for PCR and a connector oligonucleotide (5′-TACTTAGACACGACACGATTTAGTTT-3′) to guide ligation of the two DNA oligonucleotides were purchased from DNA technology (Risskov, Denmark).

### Conjugation of Antibodies

Antibodies were conjugated according to previously published protocol. 36 Briefly, 20 µg antibodies were activated with 2 µL 4 mM sulfo-SMCC (Thermo Fisher Scientific), diluted in DMSO, and incubated for 2 hours at room temperature. When 1 hour was left of the incubation time, the oligonucleotides (PLA probes) were reduced. Three microliters (100 µM stock) for each antibody were reduced with 3 µL DTT (stock 100 mM freshly dissolved in phosphate-buffered saline [PBS]/5 mM EDTA). PLA probes were incubated 1 hour at 37°C. Excess SMCC and DTT were removed by Zeba spin desalting columns with 7K cutoff (Thermo Fisher scientific) according to instructions from the manufacturer. The antibodies were then split into two aliquots and mixed with either of the two oligo arms and incubated 30 minutes at room temperature. Finally, the conjugates were dialyzed against PBS overnight at 4°C. Dialyzed antibodies were diluted to a final concentration of 500 nM in PBS supplemented with 0.1% bovine serum albumin (BSA) and 0.05% NaN _3_ and stored at 4°C.

### Solid-Phase Proximity Ligation Assay for Measurements of EVs in Plasma

Five microliters of biotinylated CT-B at a concentration of 1 mg/mL (Thermo Fisher Scientific) or 100 μL 2 μM in house biotinylated lactadherin was immobilized on 100 μL washed MyOneStreptavidin T1 dynabeads (Thermo Fisher Scientific) diluted to a final volume of 200 μL with PBS/0.1% BSA. Beads were incubated for binding of capture agent with rotation at room temperature for 1 hour. The beads were then washed twice with 500 µL PBS/0.05% Tween-20 and reconstituted in PBS with 0.1% BSA. All the washing steps were done using the DynaMag-96 Side magnet (Thermo Fisher Scientific).

Dynabeads with lactadherin or CT-B were vortexed and 1 µL for each reaction was added in a microcentrifuge tube, beads separated on magnet, and the storage buffer removed. Then 5 µL PLA buffer (PBS, 0.1% BSA, 0.05% tween-20, 100 nM Goat IgG, 0.1 µg/µL salmon sperm DNA, and 5 mM EDTA)/reaction was added to the beads and 5 µL bead suspension distributed into each well of a 96-well PCR plate. Plasma samples were then diluted 1–10 × (TF measurement) or 100 × (CD41 CD61 measurement) in PLA buffer. Diluted plasma samples (45 µL) were thereafter added and the plate was incubated with rotation for 1.5 hours at room temperature.

After incubation, samples were washed twice and 50-µL probe conjugated antibodies (CD41 and CD61 antibodies conjugated to PLA probe 1 and PLA probe 2, respectively, for platelet-EV detection; TF polyclonal antibody conjugated to either PLA probe 1 or PLA probe 2) were added at a final concentration of 500 pm . Samples were incubated 1.5 hours on rotation at room temperature and then washed three times. To remove background, samples were moved to a new 96-well plate. PCR and ligation mix was prepared by the addition of 0.01 U/µL T4 DNA ligase (Thermo Fisher Scientific) and 0.08 mM ATP to the SYBR Select master mix (Applied Biosystems, Thermo Fisher Scientific). Primers and splint were also added, at a final concentration of 0.1 µM each. The mix (50 µL) was added to each sample and the plate was incubated for 5 minutes at room temperature before the start of the qPCR program with an initial incubation step for 2 minutes at 95°C followed by 40 cycles of 15 seconds at 95°C and 1 minute at 60°C. Samples were analyzed in duplicates or triplicates. The cycle threshold (CT) values were measured in the exponential phase of the amplification, where amplification is most efficient and therefore quantification is least affected by reaction-limiting conditions. For analysis, the CT values were linearized (2^-CT) and related to background controls. To each plate and antibody combination, samples with EV-free plasma and/or plasma-free samples were included for background control.

### Flow Cytometry

Antibodies and lactadherin or annexin V were mixed to a volume of 50 µL with HBS (with 5 mM Ca ^2+^ in case of annexin V inclusion) and centrifuged at 18,000 × *g* for 5 minutes to remove antibody aggregates. After centrifugation, the antibody solution was transferred to new tubes and mixed with 30 µL plasma sample and incubated for 20 minutes at room temperature for staining. Samples were then diluted with 240 µL HBS (with 2.5 mM Ca ^2+^ if annexin V was included) and 30 µL of 3.17 µm AccuCount Blanc particles (Spherotec Inc, Lake Forest, Illinois, United States) for volume control. Samples were then analyzed within 30 minutes using a Navios or a CytoFLEX flow cytometer (Beckman Coulter). The Navios flow cytometer was set to detect both large (0.5–1 µm) and small (0.3–0.5 µm) EVs using Megamix-Plus FSC beads (Biocytex, Marseille, France). 29 42 The CytoFLEX was configured to detect EVs using the 405-nm violet laser. Gates for EVs were set using polystyrene beads sized 0.1 to 0.9 μm in diameter (Megamix-Plus FSC and Megamix-Plus SSC, Biocytex) and FSC/VioletSSC scatter plot according to instructions from Beckman Coulter (Spittler, Set-up of the CytoFLEX for EV measurement, Beckman Coulter).

### TF Procoagulant Activity Assay

The PCA of TF on the surface of EVs was assessed using a direct activity assay that measures the ability of EVs to convert FX to activated FXa in the presence of FVIIa as described previously. 43 Plasma (50 µL) was incubated for 1 hour with 1 µL lactadherin–bound T1 beads (described previously), and then plasma was separated from beads using a magnet and beads were washed twice with 200 µL HBS with 0.1% bovine serum albumin (HBSA). The beads were resuspended in 50 µL HBSA containing 10 nM FVIIa and 300 nM FX and incubated for 2 hours at 37°C. FXa generation was stopped by the addition of 25 µL 25 mM EDTA/HBSA, and then 25 µL FXa substrate (S-2765, Chromogenix, Orangeburg, New York, United States) was added and the samples incubated for 15 minutes at 37°C. The beads were removed using a magnet and the supernatant was pipetted to a 96-well plate and absorbance at 405 nm was measured using a spectrophotometer. The samples were measured in duplicates and a standard curve between 0 and 100 pg/mL was created by using relipidated human TF (Dade Innovin, Siemens, Erlangen, Germany).

### Calibrated Automated Thrombogram

The calibrated automated thrombogram (CAT) assay was used to analyze thrombin generation and TF activity in plasma samples as previously described. 31 Fluorescence was measured using a 96-well plate fluorometer (Fluoroscan Ascent, Thermo Fisher Scientific). Briefly, 80 µL of plasma was mixed with 20 µL of HEPES buffer supplemented with BSA (20 mM HEPES, 140 mM NaCl, 5 mg/mL BSA, pH 7.4) to measure thrombin generation. In parallel with the test samples, thrombin calibrator (Thrombinoscope) was run. The samples were run in duplicates and fluorometric measurements were performed after automated addition of 20 µL FluCa-kit. No exogenous TF or phospholipids were added and thrombin generation was followed for 120 minutes. The lag time before the start of thrombin generation has previously been shown to depend on TF activity. 31 44

### Statistics

Statistical analyses were performed in GraphPad Prism and differences between experimental groups were analyzed with paired or unpaired two-tailed Student's *t* -tests. Correlation between measurements was studied using Pearson's correlation. Results were considered significant when *p* < 0.05.

## Results

### Evaluation of the SP-PLA Method for the Detection of Platelet-Derived EVs in Plasma

**Fig. 1:**
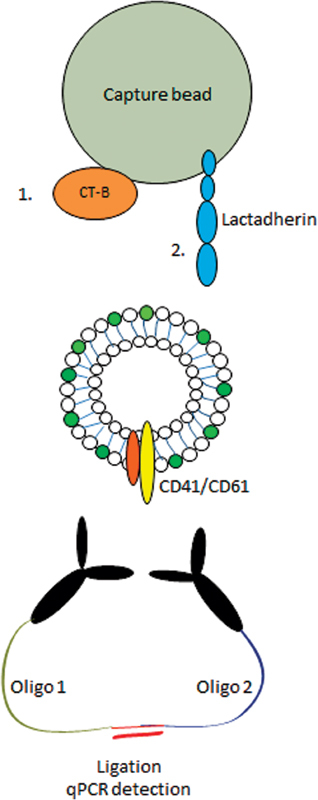
SP-PLA method for detecting EVs. CT-B or lactadherin is bound to capture beads. Capture beads with affinity reagent are then incubated with plasma diluted in PLA buffer to capture extracellular vesicles. After capture, the beads are washed and incubated with antibodies detecting CD41 or CD61 conjugated to two different oligonucleotides, PLA probes 1 or 2, to detect the CD41/CD61 integrin which is present on the platelet surface as well as on platelet-derived EVs. When the two antibodies are bound in proximity, PLA probes can be ligated and the ligation product serves as a template for a PCR reaction. Then detection can subsequently be done sensitively using quantitative PCR.

**Fig. 2:**
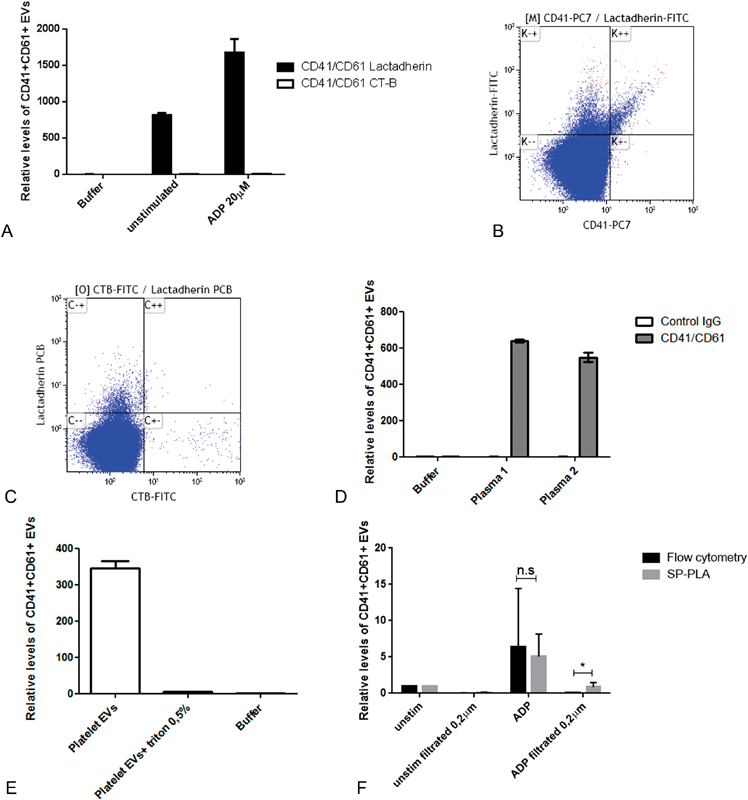
Detection of platelet-derived EVs using SP-PLA. ( **A** ) Detection of CD41/CD61+ EVs in plasma (diluted 1:100 in PLA buffer) using SP-PLA after lactadherin capture (black bars) and CT-B capture (white bars). Levels relative to the negative control with only PLA buffer (set to 1) are shown. Error bars represent standard deviation ( *n* = 2). ( **B** ) Plasma from ADP-stimulated whole blood was analyzed by flow cytometry using a Navios from Beckman Coulter with the gate set on 0.3–0.9 μm particles defined by polystyrene beads. One representative flow cytometry analysis (dot plot) of lactadherin-FITC and CD41-PC7–positive events is shown. ( **C** ) Representative flow cytometry analysis (dot plot) of CT-B-FITC and lactadherin PCB. ( **D** ) Mouse control IgG or CD41 and CD61 antibodies were applied in SP-PLA and signals from EVs in plasma (diluted 1:100) from two individuals are shown relative to negative control with only PLA buffer (set to 1). Error bars represent standard deviation from replicates ( *n* = 2). ( **E** ) Purified platelet EVs were lysed or not with 0.5% triton-X-100 for 5 minutes prior to SP-PLA analysis. Signal in un-lysed and triton lysed samples are shown as levels relative to negative control with only PLA buffer (set to 1). Error bars represent standard deviation from duplicates. ( **F** ) Whole blood was activated or not with 20 μM ADP for 2 hours and plasma was generated. The plasma samples were filtered or not with 0.2 μm filters and the levels of platelet EVs (CD41/CD61 positive) were measured with SP-PLA and high-sensitivity flow cytometry using a Navios from Beckman Coulter, with the gate set by polystyrene beads on 0.3–0.9 μm particles. Levels relative to unstimulated control are shown. Error bars represent standard deviation from three individual experiments ( *n* = 3).

To detect different EV populations in plasma, we applied the SP-PLA as illustrated in Fig. 1 . To evaluate the method, a series of experiments were conducted on platelet-derived EVs (CD41 + /CD61 + ). Lactadherin, but not CT-B, readily captured platelet-derived EVs in plasma from both unstimulated and ADP-stimulated whole blood ( Fig. 2A ). Flow cytometric analysis further confirmed that almost all of the detectable CD41-positive EVs in plasma were indeed positive for PS ( Fig. 2B ) and that CT-B and lactadherin seemed to stain different EV populations ( Fig. 2C ). Exchanging the antibodies used for the detection with a mouse control IgG completely removed the PLA signal demonstrating the requirement of specific antibodies for detection of EVs ( Fig. 2D ). Treating purified platelet EVs with 0.5% triton X-100 prior to analysis also depleted the signal, showing that the assay is detecting vesicles ( Fig. 2E ).

To compare high-sensitivity flow cytometry (Navios, Beckman Coulter) with the SP-PLA method, plasma from unstimulated whole blood and plasma from whole blood stimulated with 20 μM ADP were analyzed before and after 0.2-μm filtration. Levels of CD41 + /CD61+ EVs relative to the respective unstimulated control before and after filtration are shown in Fig. 2F . The plasma filtration almost completely depleted the signal from the flow cytometer, whereas EVs were still detectable with the lactadherin SP-PLA method. The mean reduction after filtration of plasma from ADP-stimulated whole blood was 79% with PLA and 96% with flow cytometry ( *p* = 0.048, *n* = 3).

**Fig. 3:**
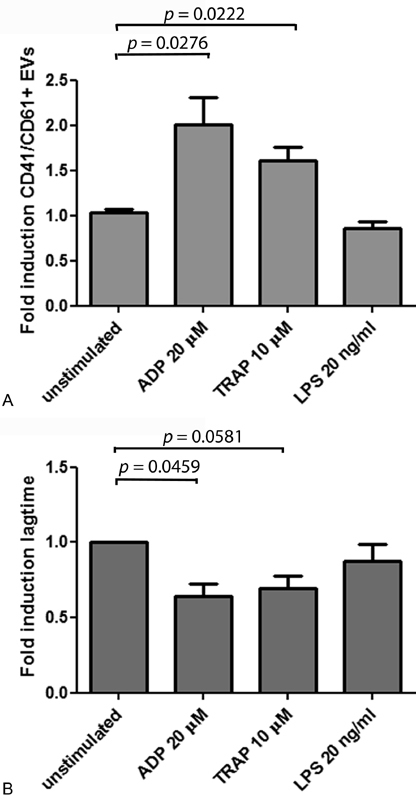
Platelet-derived EVs in plasma samples measured by SP-PLA and a thrombin generation assay. Whole blood was stimulated or not with 20 μM ADP, 10 μM TRAP, or 20 ng/mL LPS in 37°C for 3 hours to generate EVs. ( **A** ) CD41/CD61+ EVs were detected with SP-PLA and the levels are shown as fold induction from the mean value of plasma prepared from unstimulated whole blood (unstimulated, set to 1). Error bars represent standard error of the mean (SEM). Differences between groups were tested with unpaired Student's *t* -test, and a *p* -value <0.05 was considered significant. ( **B** ) Thrombin generation was studied in the same samples using calibrated automated thrombogram (CAT). Fold induction of the lag time in plasma from unstimulated whole blood is shown. Error bars represent SEM from three individual experiments. Differences between groups were tested with a paired, two-tailed Student's *t* -test. A *p* -value < 0.05 was considered significant.

In addition to treatment with ADP ( Figs. 2A and 3A ), whole blood was also stimulated with TRAP and LPS as indicated in Fig. 3A . ADP and TRAP increased the formation of platelet-derived plasma EVs, whereas LPS did not. The ADP and TRAP treatments also reduced the lag time for thrombin generation in the CAT assay, showing that the stimulated sample was procoagulant ( Fig. 3B ).

### Determination of Limit of detection Score and Assay Variability

**Fig. 4:**
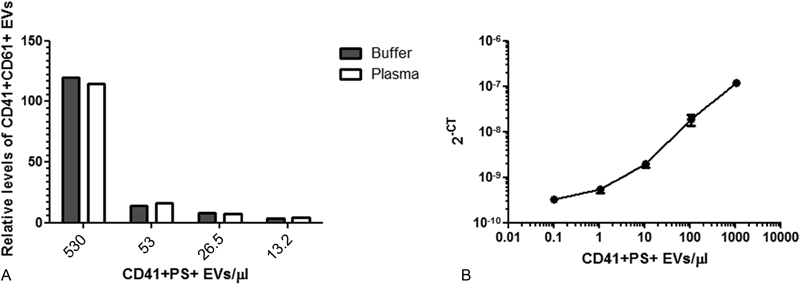
Detection of spiked platelet-derived EVs. ( **A** ) Platelet EVs were counted by flow cytometry (Navios, Beckman Coulter) and spiked at different concentrations either in PLA buffer or in EV-free plasma (diluted 1/100). The PLA signals relative to background (only buffer) after lactadherin capture and CD41/CD61 detection from a representative experiment ( *n* = 1) is shown. ( **B** ) To determine the LOD score, washed platelet-derived EVs were counted by flow cytometry (Navios). The EVs were then spiked in PLA buffer to create a dilution series (1:10) and detected with SP-PLA CD41/CD61. Error bars represent standard deviation ( *n* = 3).

To further demonstrate the performance of SP-PLA, we spiked washed platelet-derived EVs into EV-free plasma or into buffer. The platelet-derived EVs were first counted using a Navios flow cytometer (Beckman Coulter) with a polystyrene bead-defined gate set on 0.3 to 0.9 μm. The platelet-derived EVs were then spiked at different concentrations either in EV-free plasma (diluted the same way as we dilute plasma for SP-PLA analysis, 1:100) or directly in PLA buffer. The obtained signals after the SP-PLA were similar for both spike-in in buffer and plasma, showing that detection of platelet-derived EVs in plasma is plausible ( Fig. 4A ).

Limit of detection (LoD) is the lowest analyte concentration likely to be reliably distinguished from the background and at which detection is feasible. To determine the LoD for SP-PLA, platelet EVs were spiked in a 10-fold dilution series in EV-free plasma diluted 1:100 with PLA buffer and detected with SP-PLA. The concentration of CD41+ PS+ EVs was plotted against the SP-PLA signal ( Fig. 4B ) and the LoD (CT value: 2 standard deviations above the background) corresponded to a measured concentration of 0.1 EVs/μL. The intra-assay variability (mean %CV calculated for technical triplicates at all five measured concentrations) was 16% and the inter-assay variability was 17.7% for 1,500 CD41+ PS+ EVs/μL and 18.3% for EV-free plasma (estimated by measuring samples in triplicates in 10 independent experiments and calculating the %CV of the means).

### SP-PLA Correlates Well with a High-Sensitivity Flow Cytometric Analysis

**Fig. 5:**
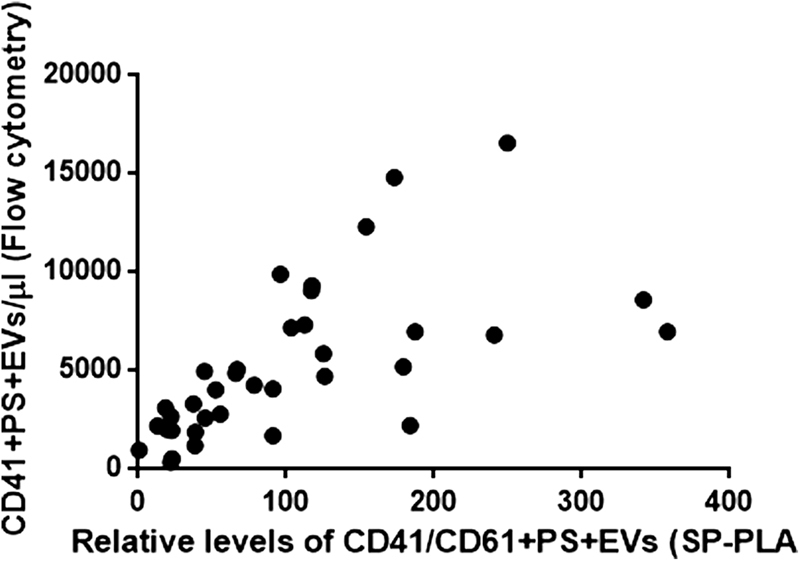
Correlation between SP-PLA and high-sensitivity flow cytometric measurements of platelet-derived EVs. SP-PLA measurements of platelet-derived EVs in plasma from REBUS patients (relative to EV-free plasma that was set to 1) were plotted against CD41 + AnnV+ EVs counted by high-sensitivity flow cytometry using the CytoFLEX (Beckman Coulter) with the gate set by polystyrene beads on 0.1–0.9 μm particles .Correlation was determined using the Pearson product-moment correlation coefficient (R = 0.65, *p* < 0.0001, *n* = 34).

Next, to compare the SP-PLA signal for platelet-derived EVs with a high-sensitivity flow cytometry assay, the CytoFLEX system (Beckman Coulter) was used according to the guidelines for EV detection provided by the manufacturer (Spittler, Set-up of the CytoFLEX for EV measurement, Beckman Coulter). The gates for EVs were set using FSC/SSC scatter plot and polystyrene Mega mix beads with the diameters 0.1, 0.16, 0.2, 0.24, 0.3, 0.5, and 0.9 μm. All bead sizes could be resolved from one another using the violet side scatter. Plasma samples from 17 patients with myocardial infarction included in the REBUS study (at inclusion and at 1-year follow-up) and 3 controls (in total 37 samples) were used and a significant correlation between the SP-PLA signal and flow cytometric measurements of platelet-derived EVs was found (Pearson's correlation [ *R* ] = 0.63, *p* < 0.0001; Fig. 5 ).

### Assay for CT-B-Captured TF+ EVs Is Specific and Detects Extracellular Vesicles

**Fig. 6:**
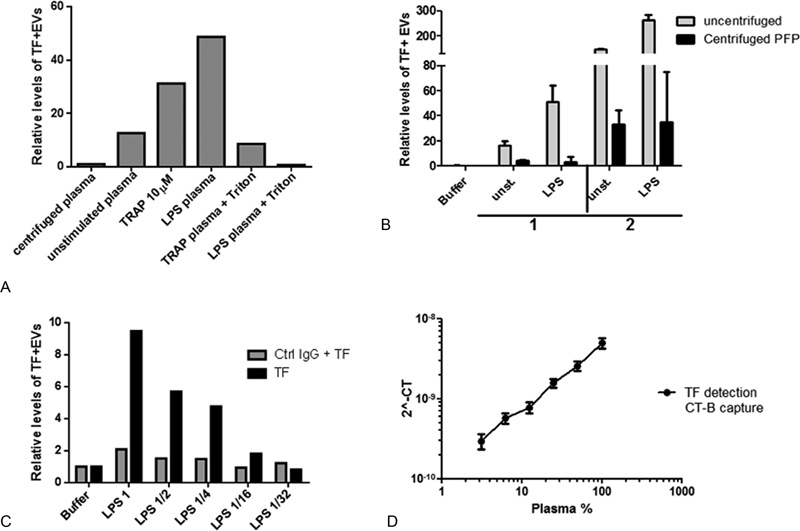
Detection of TF+ EVs using SP-PLA and CT-B capture. ( **A** ) The levels of TF+ EVs was measured with SP-PLA by using CT-B capture in unstimulated plasma or in plasma from TRAP or LPS-stimulated whole blood with or without triton-X100 treatment. Levels relative to EV-free plasma are shown. ( **B** ) The levels of TF+ EVs were measured with SP-PLA in plasma samples from unstimulated and stimulated whole blood from two individuals before (white bars) and after (black bars) centrifugation at 20.000 × *g* for 60 minutes. Levels relative to buffer alone are shown. Error bars represent standard deviation from technical replicates ( *n* = 2). ( **C** ) Levels of TF+ EVs were measured by SP-PLA using the TF polyclonal goat antibody conjugated to both PLA probes as usual (black bars) or by using the TF polyclonal goat IgG (conjugated to probe 1) together with a control goat IgG (conjugated to probe 2) (gray bars). The levels relative to signal in buffer alone is shown. ( **D** ) Levels of TF+ EVs were measured with SP-PLA using CT-B capture in plasma from LPS-stimulated whole blood, diluted 1:2 in a dilution series. The plasma concentration is plotted against linearized CT values. Error bars represent standard deviation of technical replicates ( *n* = 3).

To test if the method would work on a less abundant EV population, we applied the SP-PLA method on TF+ EVs. For this purpose, we first captured the EVs with CT-B and used a TF polyclonal antibody conjugated to PLA probes for detection. The TF+ EVs were captured by CT-B in 5 µL plasma, and the total levels of EV TF (that can reflect EV numbers and/or TF expression on EVs) were increased after stimulation of whole blood with TRAP, LPS ( Fig. 6A ), and ADP (data not shown). Treatment with 0.5% Triton X-100 for 10 minutes prior to the SP-PLA ( Fig. 6A ) and centrifugation of LPS plasma at 20,000 × *g* for 30 minutes ( Fig. 6B ) both resulted in lower levels of TF+ CT-B+ EVs, indicating that the assay detected EVs. To control for the specificity of antibodies, one of the detection antibodies was exchanged for a mouse control IgG antibody. As demonstrated in Fig. 6C , the signal disappeared with this antibody pair which means that a specific detection was recorded by our TF antibody pair. A 10-fold dilution series of plasma with buffer furthermore decreased the SP-PLA signal proportionally ( Fig. 6D ).

### Detection of PS+ TF+ EVs in Plasma

**Fig. 7:**
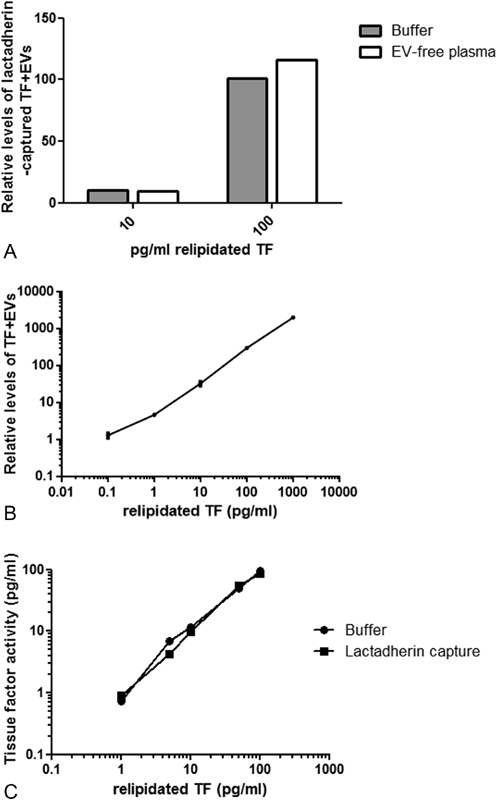
Detection of PS+ TF+ EVs using SP-PLA and lactadherin capture. ( **A** ) Relipidated TF was spiked into buffer or EV-free plasma and detected with SP-PLA after lactadherin capture. Levels relative to control with EV-free plasma (set to 1) are shown. ( **B** ) Relipidated TF was spiked at different concentrations in PLA buffer and TF+ EVs were detected with SP-PLA after lactadherin capture. Error bars represent standard deviation from technical replicates ( *n* = 2). ( **C** ) TF PCA of relipidated TF was measured using a direct activity assay after capture directly in the buffer or after lactadherin capture.

In contrast to CT-B, we were unable to capture TF+ EVs with lactadherin in 5 µL plasma in the stimulated whole blood from healthy individuals. However, a spike-in of relipidated TF could be detected in our EV-free plasma with the same efficiency as in buffer ( Fig. 7A ). Upon TF spike-in, the SP-PLA for TF+ EVs, captured by lactadherin, had a detection range of at least 4 logs and a LoD score of 0.5 pg/mL ( Fig. 7B ). The intra-assay variability (mean %CV of technical triplicates at all the measured concentrations) was 11%. Interassay variability (% CV of mean value) measured for 10 and 100 pg/mL in five independent experiments was 7 and 17%, respectively.

### TF PCA of Lactadherin-Captured TF+ EVs

The PCA of TF on the surface of EVs was determined using a direct activity assay measuring the ability of EVs to convert FX to FXa in the presence of FVIIa. 43 The measured levels of relipidated TF were similar after capture with 1 μL lactadherin T1 beads as if measured directly in the buffer ( Fig. 7B ).

## Discussion

In this report, we show that EVs can be accurately measured in only a few microliters of plasma using a qPCR machine, standard equipment in most laboratories which is cheap and easy to handle as compared with a flow cytometer. With PCR, very few templates can be amplified and detected quantitatively using a real-time quantitative approach, resulting in high sensitivity. Based on the SP-PLA technique, we have developed an assay that specifically and sensitively detect platelet-derived EVs (CD41 + /CD61+ and PS + ) as well as TF+ EVs in small amounts of plasma. Treatment with detergent or centrifugation of the samples depleted the SP-PLA signal, showing that the method detects vesicular structures. In addition, by filtering the plasma with a 0.2-μm filter, we completely removed the flow cytometry count using the Navios instrument from Beckman Coulter, whereas the CD41/CD61 EVs were still detectable by SP-PLA. This indicates that our method can detect vesicles smaller than 0.2 μm.

We also compared SP-PLA platelet EV detection using only 0.5 µL of plasma with the new CytoFLEX system (Beckman Coulter), which can detect polystyrene beads as small as 0.1 μm in diameter. We found that there was a significant correlation between the two analysis methods ( *R* = 0.6475; *p* ≤ 0.0001). The values tended to be higher with SP-PLA detection in some samples, which may be explained by the fact that CytoFLEX measures EVs per microliter, whereas SP-PLA measures total amount of antigen on all EVs. Samples with a higher degree of large EVs with high expression of CD41/CD61 might thus increase the SP-PLA signal without affecting the count in the CytoFLEX. As stated, with the SP-PLA also EVs below even the detection limit of the CytoFLEX may be measured, which also could contribute to the discrepancies in the measurements.

Measuring the total antigen levels on the EV surface (as we do with SP-PLA) and counting EVs (as with flow cytometry) are two different approaches which provide different information about the EVs and the methods could complement each other. Flow cytometry can separate the subsets of EVs based on scattering properties and fluorophores counting the number of EVs. The SP-PLA method measures the total EV surface antigen levels in a sensitive manner and provides additional information to the amount of EVs found by flow cytometry. Since the surface levels of adhesion molecules or tissue factor are relevant for functionality such as activation of coagulation, the total antigen levels might be clinically relevant for certain EV populations. For example, platelet-derived EVs with high levels of adhesion molecules adhere to sites of thrombotic activity, whereas erythrocyte-derived EVs, which lack adhesion receptors, are found in the plasma. 45 In this article, we have focused on the platelet-derived EVs as well as those carrying tissue factor. However, there are populations of EVs from other cell types found in plasma with different functions and the SP-PLA method can be further developed to detect other EV populations, in particular those with small size and/or low antigen levels that may be difficult to detect with flow cytometry. In addition to solving the problem with measuring EVs in the full-size range, SP-PLA and other proximity-based technologies such as 4 PLA, ExoPLA, and multiplex extension assays 14 33 46 offer several more advantages over traditional flow cytometry. As exemplified by our plasma SP-PLA method, these new techniques offer higher specificity as three affinity reagents are necessary for detection. PLA has also been shown to give higher sensitivity than ELISA. 47 These are clear advantages for the analysis of small EVs and/or EVs with low levels of surface protein. Multiplex analysis is also possible 46 enabling parallel analysis of several EV populations in a sample.

To capture EVs, we used two capture agents, lactadherin and CT-B. CD41 + /CD61+ EVs (platelet-derived EVs) were found in the EV population that binds to lactadherin, whereas TF+ EVs were primarily found in the CT-B-bound population in plasma from in vitro stimulated blood donated from healthy individuals. Flow cytometry measurement confirmed that CD41+ EVs are PS positive, and that CT-B and lactadherin, at least to a large extent, measure different vesicle populations. This is in agreement with a recent report investigating biomarkers for preeclampsia. The authors of this report found that distinct vesicles were captured by annexin V or CT-B. 39 CT-B binds to ganglioside GM1 in the plasma membrane. GM1 is enriched in lipid rafts and caveolae 48 which, among other things, appears to favor membrane blebbing and EV formation. 49 Release of TF+ EVs has also been associated with lipid rafts 50 and we found that TF+ ganglioside GM1+ EVs are released upon stimulation with ADP, TRAP, and LPS. The TF+ EVs were also depleted by triton-X-100 treatment or centrifugation and were detected only in 5 µL plasma using the SP-PLA method. LPS stimulation of whole blood has previously been shown to upregulate TF in monocytes and TF PCA has been detected on EVs in centrifuged plasma derived from LPS-stimulated whole blood. 44 51 52 53

PS+ TF+ EVs are present at very low concentrations in plasma and initially we did not find PS+ TF+ EVs using the SP-PLA analysis with lactadherin capture. However, spike-in of relipidated TF was captured with lactadherin and detected with SP-PLA generating a similar signal to background ratio in our EV-free plasma pool as in PLA buffer, showing that the assay works well also for PS+ EVs. PS+ TF+ EVs were detected down to concentrations of below 1 pg/mL, which is similar to a direct TF PCA assay. 43 By using a previously described direct TF PCA assay, 43 we also found that lactadherin-captured relipidated TF spike-in generated a similar activity level as if activity was measured directly in the buffer, showing that the capture of PS+ EVs is efficient.

The levels of TF+ EVs in plasma are usually low and flow cytometric analysis and ELISA have been shown to correlate poorly with activity. 51 A possible reason for a discrepancy between levels of TF+ EVs and TF PCA might be that TF exists both in an active and an encrypted conformation and only the former is procoagulant. 54 Functional assays can be used to detect TF+ EVs, such as a direct TF PCA assay or CAT assay. 43 44 Then only the active/procoagulant TF, which might be more clinically interesting, is accounted for. However, the presence of PS− TF+ EVs has been reported previously 55 56 and monocytic EVs have been shown to fuse with activated platelets to initiate coagulation, 50 implying that EVs lacking PS can still become procoagulant under certain circumstances. The measurement of TF PCA in plasma often includes a high-speed centrifugation step or capture with annexin V (affinity which is Ca ^2+^ dependent) to separate the EVs from plasma. 43 51 52 Using SP-PLA with lactadherin capture, we can detect the TF+ PS+ procoagulant EVs sensitively and without a need for centrifugation or Ca ^2+^ .

In summary, our novel SP-PLA method with lactadherin and CT-B as capture reagents, using only 0.5 to 45 µL sample, can detect different types of EVs with high specificity and sensitivity, providing information of the total antigen level and has the potential to be an attractive complement method to flow cytometric analysis of preclinical and clinical samples. With this SP-PLA, the smallest EVs can also be analyzed. Techniques for measuring EVs have rapidly improved during the last years, and this development provides hope that it will be possible to study EVs more efficiently and accurately in the future, with the purpose to identify populations of EVs that might be valuable for clinical assessment of various diseases.
